# Transcriptomic analysis reveals critical genes for the hair follicle of Inner Mongolia cashmere goat from catagen to telogen

**DOI:** 10.1371/journal.pone.0204404

**Published:** 2018-10-24

**Authors:** Rui Su, Yixing Fan, Xian Qiao, Xiaokai Li, Lei Zhang, Chun Li, Jinquan Li

**Affiliations:** 1 College of Animal Science, Inner Mongolia Agricultural University, Hohhot, Inner Mongolia, China; 2 Key Laboratory of Animal Genetics, Breeding and Reproduction, Inner Mongolia Autonomous Region, Hohhot, China; 3 Key Laboratory of Mutton Sheep Genetics and Breeding, Ministry of Agriculture, Hohhot, China; 4 Engineering Research Center for Goat Genetics and Breeding, Inner Mongolia Autonomous Region, Hohhot, China; 5 College of Animal Science, Inner Mongolia University for Nationalities, Tongliao, Inner Mongolia, China; University of Illinois, UNITED STATES

## Abstract

There are two main types of hair follicle in Inner Mongolia Cashmere goats, the primary hair follicle (PHF) producing hair fibers and the secondary hair follicle (SHF) producing cashmere fibers. Of both fibers from cashmere-bearing goats in Aerbasi, Inner Mongolia, the timing of cyclical phases for the cashmere have been well clarified but hair fibers have been less noticeable. Herein, we evaluated transcriptome of PHF and SHF from the same three goats in Aerbasi at the catagen- and telogen phase of cashmere growth. We totally found 1977 DEGs between PHFs at the telogen and catagen phases of SHF, 1199 DEGs between telogen- and catagen SHF, 2629 DEGs between PHF at the catagen phase of SHF and catagen SHF, and 755 DEGs between PHF at the telogen phase of SHF and telogen SHF. By analyzing gene functions based on GO and KEGG database, we found that the DEGs have functions in muscle contraction and muscle filament sliding between catagen- and telogen SHF, indicating that arrector pilli muscles might play a role on the transition from catagen to telogen. Moreover, considering that the enriched GO and KEGG categories of the DEGs between PHF and SHF, we suggested that part of PHF might rest in their own anagen phase when SHF are at catagen, but PHF might enter into the telogen phase at SHF’s telogen. Finally, we highly recommended the several potential genes acting as the regulators of the transition between growth phases including *IL17RB* and eight members of ZNF. These results provide insight into molecular mechanisms on the transition of SHF from catagen to telogen together with PHF’s growth situation at SHF’s catagen and telogen in Inner Mongolia Cashmere goats.

## Introduction

**Fig 1:**
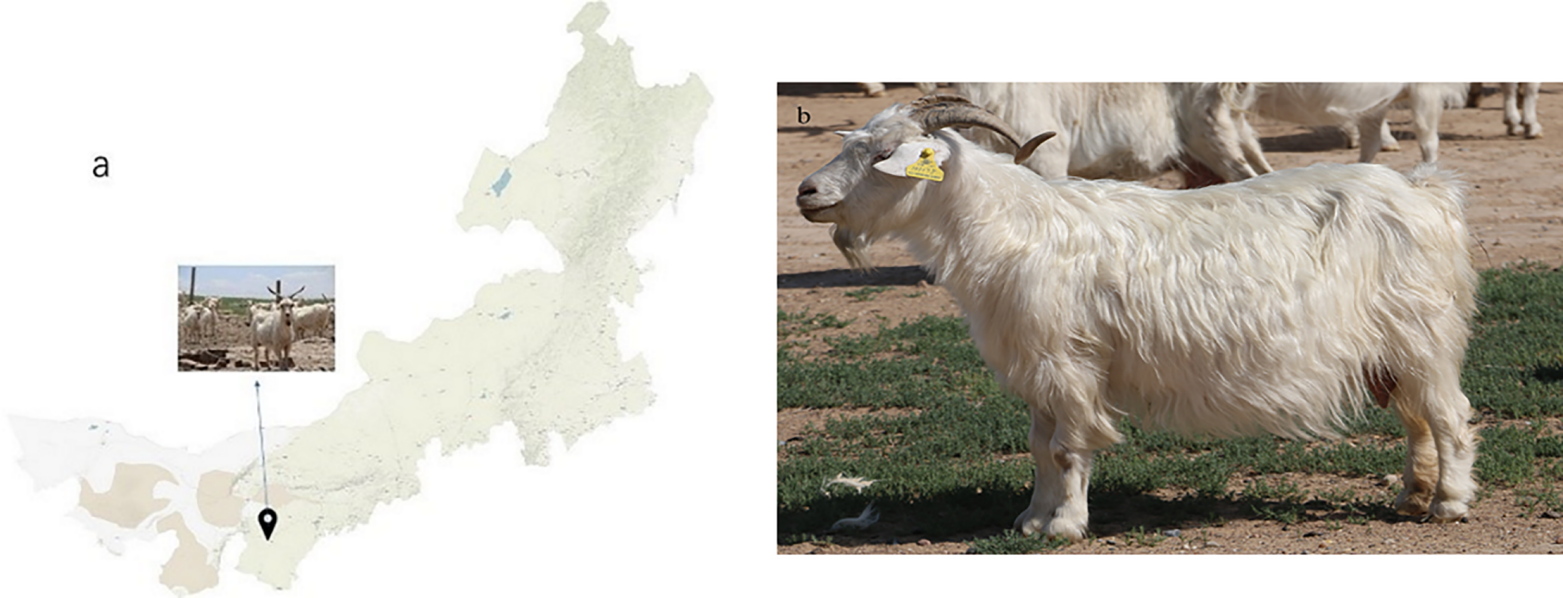
(a) is Inner Mongolia Autonomous Region map and the location is Otog Banner where Inner Mongolia Cashmere goat strain line of Aerbasi mainly distributed. (b) is a female goat of this breed.

Hair follicles on animal skin execute many useful biology function except for producing hair, such as immune defense and grease secretion, etc. There are two main types of hair follicle in goats, the primary hair follicle (PHF) producing hair fibers and the secondary hair follicle (SHF) producing cashmere fibers. Cashmere is finer, stronger, lighter, softer, and more insulating than sheep wool, surely it looks as a luxury fiber[1]. As a major province of raw cashmere in China, Inner Mongolia bears three types of Cashmere goats—Erlangshan, Aerbasi and Alashan; these Cashmere goats are mainly distributed in the northwest of Inner Mongolia Plateau (Fig 1). They are superior in the production of cashmere with diameter less than 16 μm.

Hair follicles is a unique, highly regenerative organ with a normal developmental cycle, which is mainly determined by the interaction of epithelial cells and dermal papilla that are generally composed of dermal mesenchymal cells [2]. A growth cycle consists mainly of three distinct stages—anagen, catagen and telogen [2,3,4]. In Cashmere goat, SHF takes approximately one year to complete a growth cycle. For Inner Mongolia Cashmere goats, the anagen phase of SHF begins from April until November, the catagen phase is from December to January, and the telogen phase is from February to March[5]. HFs help cashmere to grow at the anagen phase, turn into apoptosis at the catagen phase and finally release cashmere at the telogen phase. When the next anagen phase comes, HFs launch a new regenerative cycle. To increase the harvest accompanying with the thinner cashmere, researchers try to find major genes and pathways, which may affect the of development and growth the cashmere by molecular biology methods.

RNA-seq is usual and convenient for researchers to study different animal phenotypes due to differentially expressed genes (DEGs), because we could get enough transcriptome data from small quantities of tissue samples[6,7]. In previous studies of hair follicle in Cashmere goats, the scientists have already found many important factors that may affect hair follicle cycle in Wnt signal transduction pathway, fibroblast growth factor (FGF) family, bone morphogenetic protein (BMP) family, Sonichedgehog (Shh) signal transduction pathway, transforming growth factor (TGF) family, Notch signal transduction pathway and so on [8,9,10]. Some genes play an activator role while other genes play roles in these pathways as the inhibitors. *β-catenin* has been confirmed as an important accelerator in Wnt signal transduction pathway, but more importantly, its activity is simultaneous with the apoptosis of hair follicle stem cells [11]. Maksim V. Plikus found that BMPs may be the long-sought inhibitors of hair growth postulated by classical experiments[12].

In this study, we used RNA-seq technology to find some important factors in SHF of Inner Mongolia Cashmere goats by comparing catagen SHF with telogen SHF. Furthermore, functional annotation analysis also helps us find some important genes that influence cashmere growth. By comparing the DEGs of PHF at the catagen and telogen phase of SHF, the growth condition of PHF became relatively clear. Our data might take insight into the molecular mechanism on the growth of SHF and PHF at the catagen- and telogen phase of SHF.

## Material and methods

### 1. Ethics statement

In this study, hair follicles were collected in accordance with the International Guiding Principles for Biomedical Research Involving Animals and was approved by the Animal Ethics Committee of the Inner Mongolia Academy of Agriculture and Animal Husbandry Sciences that is responsible for Animal Care and Use in the Inner Mongolia Autonomous Region of China. In our study, no specific permissions were required for these activities and the animals did not involve endangered or protected species.

### 2. Hair follicle samples preparation for RNA-seq and qRT-PCR validation

Three 3-years-old female Aerbasi Inner Mongolia Cashmere goats from goat stud farm (Aerbasi White Cashmere Goat Breeding Farm, Erdos, Inner Mongolia) were used in this study for RNA-seq. At the dorsal side of goats, we collected PHF and SHF samples by pulled out hair and cashmere from the root of HFs in SHF’s catagen (in mid-January) and its telogen (in mid-March), respectively; and then put them in liquid nitrogen as soon as possible and finally stored in -80°C refrigerator for a long term. About 50 to 100 hair follicles of three cashmere-bearing goats were collected as a sequencing sample. Six female goats, which come from the same group at same age as the RNA-seq goats, were used in qRT-PCR.

### 3. Construction of RNA library, sequencing and quality control

Total RNA was extracted and test the concentration. Each RNA sample needs over than 2 μg and RIN (the integrity of RNA) value larger than six. We mixed three cashmere goats total RNA and then constructed a library. In hence, four libraries were constructed. After high throughput sequencing, we used Fast-QC to measure the quality of sequence data, including base quality distribution, GC%, content of PCR duplication and frequency of kmer.

### 4. Mapping reads to reference genome and gene structure analyze

After filtration, we mapped the clean reads to goat gene sequences (http://goat.kiz.ac.cn/GGD/download.htm) and goat reference genome sequences (http://goat.kiz.ac.cn/GGD/download.htm) by MapSplice[4,13]. Then calculated the gene structure, exons, introns and intergenic regions.

### 5. Expression level analysis

RPKM means the genes expression and calculating bases on the reads counts mapped to this gene and gene length[14]. The different expression genes between catagen and telogen was calculated by EBSeq (Log2FC>1 or Log2FC<-1, FDR<0.05)[15].

### 6. GO and KEGG pathway analysis

We analyzed genes function by Gene Ontology (GO), an international gene functional classification system, on three categories including biological process, molecular function and cellular component[4,16]. We used Fisher formula to exam the *P*-Value of each GO analysis and blasted result onto human and mouse reference genomes (*P*-Value<0.01 represent gene function is significance). Different expression genes (DEGs) were annotated by Kyoto Encyclopedia of Genes and Genomes (KEGG) database (http://www.genome.jp/kegg) to identify the pathway enrichments[17,18,19].

### 7. qRT-PCR to validate RNA-seq data

To validate the veracity of RNA-seq data and genes expression level, we used quantitative real time PCR in this experiment. First, we extracted total RNAfrom catagen’s- and telogen’s SHF from six goats. Second, we synthesized cDNA from mRNA. Then, the primers we used was designed and synthesized depending on the mRNA sequences published on NCBI database. QRT-PCR was used SYBR GreenⅡ. In the qRT-PCR, β-actin was acted as internal reference and DEGs expression level was calculated by 2^-ΔΔct^. The primer sequences information was shown in Table 1.

**Table 1:** The genes primer sequences information.

gene name	Reference Sequence	primer sequence	TM (°C)
*IL17RB*	XM_005695840.1	F: CTCCCAAGACCTGTTCCACC	61
		R: GCGCACTGTAGCTGTCTTTG	
*β-actin*	NM_001314342.1	F: GGCAGGTCATCACCATCGG R: CGTGTTGGCGTAGAGGTCTTT	60

Reaction system of qRT-PCR was totally 20μl, including SYBR Green Ⅱ 10μl, primer F 0.5μl, primer R 0.5μl, ROX 0.4μl, cDNA 2μl and H2O 6.6μl. The condition of the reaction was departed into amplification stage and dissociation stage. Amplification stage includes 95°C 30s for 1 cycle, 95°C 5s and annealing temperature (TM) 34s for 40 cycles, dissociation stage includes 95°C 15s, 60°C 60s and 95°C 15s. Result were analyzed by SPSS 17.0.

## Result

**Fig 2:**
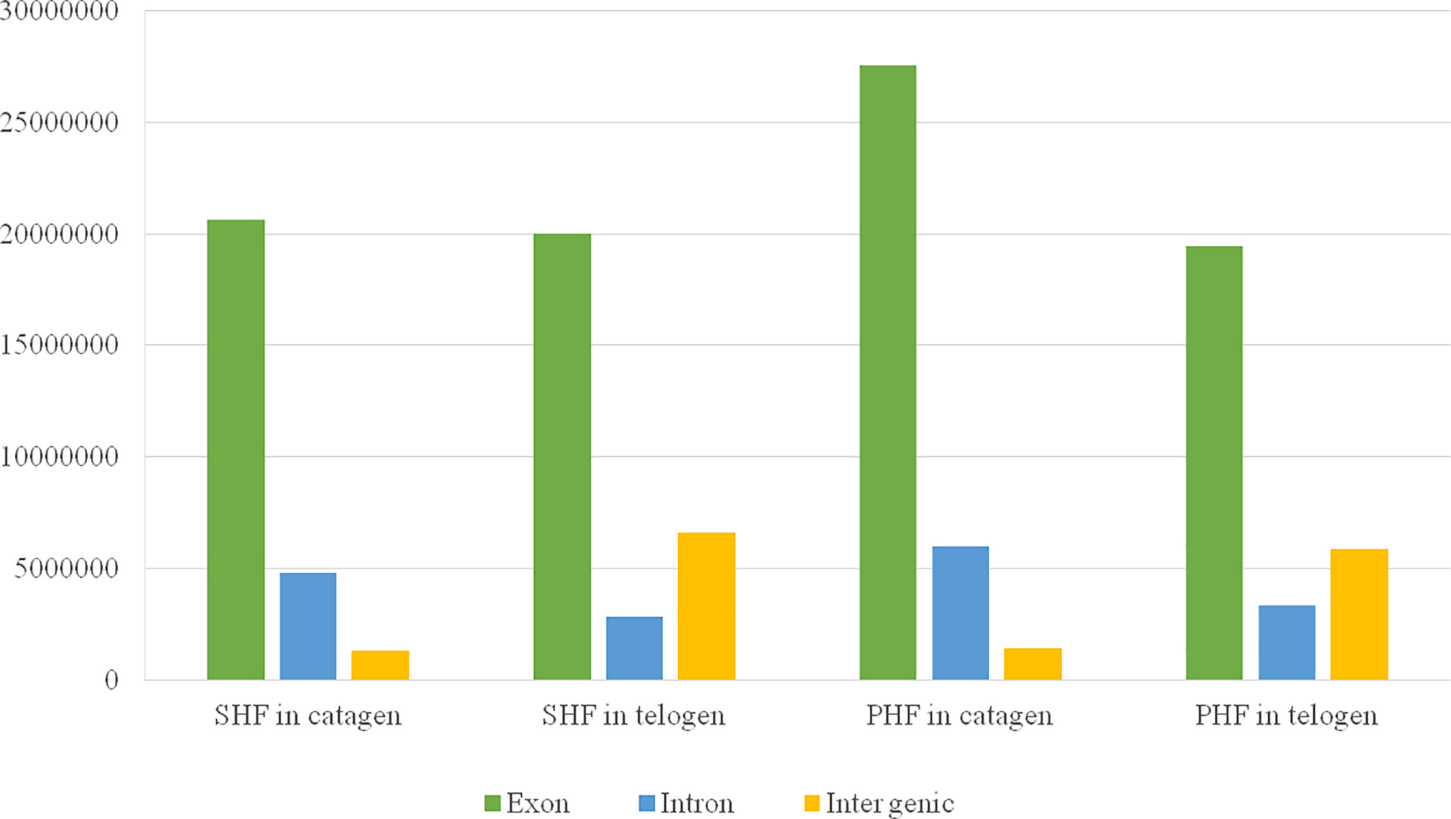
Gene structure in catagen and telogen of SHF and PHF. The number of exons of SHF in catagen and telogen respectively are 20617193 and 19999609, introns are 4822842 and 2832110, intergenic regions are 1309521 and 6618361. However, the number of exons of PHF in catagen and telogen respectively are 27541761 and 19455711, introns are 5950502 and 3337148, intergenic regions are 1387033 and 5853974. From the figure, we can find that in these four samples, the largest number is exon. But for intron and intergenic regions, there are some differences between catagen and telogen. In catagen, the number of intron is higher than intergenic regions, while in telogen is opposite.

To gain insight into the growth of hair follicles at SHF’s cyclic phases, we quantified the whole-genome transcriptomes of primary- or secondary hair follicles at the catagen and telogen phases of SHF in 3 female Inner Mongolia Cashmere goats. In total, we obtained over 25 million clean reads from each library after trimming, adaptor sequences, low quality reads and multiple mapped reads (Table 2). Gene structure was showed in Fig 2. The clean reads were mapped to 22175 genes annotated in the goat reference genome (*Capra Hircus 1*.*0*). By comparing the RNA-seq data, we then identified DEGs in the following four groups including (i) PHF at the telogen and catagen phases of SHF, (ii) telogen- and catagen SHF, (iii) PHF at the catagen phase of SHF and catagen SHF, and (v) PHF at the telogen phase of SHF and telogen SHF (Table 3). Using the thresholds of false discovery rate (FDR) < 0.05 and difference ratio of RPKM (reads per kilobase of exon per million fragments mapped) > 2, we defined 1977 DEGs between PHF at the telogen and catagen phases of SHF, 1199 DEGs between telogen and catagen SHF, 2629 DEGs between PHF at the catagen phase of SHF and catagen SHF, and 755 DEGs between PHF at the telogen phase of SHF and telogen SHF. When focusing on the characterized transcripts, the number of DEGs among the four groups arrays in ascending order as follows: 1783 DEGs between PHF at the catagen phase of SHF and catagen SHF, 1092 between PHF at the telogen and catagen phases of SHF, 552 between telogen and catagen SHF, and 422 between PHF at the telogen phase of SHF and telogen SHF. It is noteworthy that, based on the number of DEGs, the largest distinction at transcription level should be between PHF at SHF’s catagen phase and catagen SHF in sharp contrast to the smallest difference between PHF at SHF’s telogen phase and telogen SHF. Interestingly, the transcriptomic changes seem to be relatively small between telogen- and catagen SHF compared with those between PHF at the catagen- and telogen phase of SHF.

**Table 2:** Mapping statistics of SHF and PHF in both catagen and telogen.

Statistics Item	SHF in catagen	SHF in telogen	PHF in catagen	PHF in telogen
All reads	39905590	38656183	40472235	38929776
Unmapped	13543655	8353891	6029692	9019823
Mapped	26361935	30302292	34442543	29909953
Mapped rate	0.661	0.784	0.851	0.768
Unique mapped	25111435	28929192	32528469	28709741
Unique mapped rate	0.629	0.748	0.804	0.737
Repeat mapped	1250515	1373099	1914088	1200212
Junction all mapped	5307898	1338926	7746160	3207050
Junction unique mapped	5304888	1337307	7742669	3205570

**Table 3:** Different expression genes identified by RNA-seq.

	PHF_telogen vs PHF_catagen		SHF_telogen vs SHF_catagen		SHF_ catagen vs PHF_ catagen		SHF_telogen vs PHF_telogen	
	upregulated	downregulated	upregulated	downregulated	upregulated	downregulated	upregulated	downregulated
Characterized genes	591	501	360	192	939	844	302	120
Uncharacterized genes	667	218	593	54	601	245	290	43

Next, we performed a GO annotation analysis for these characterized DEGs in the four groups. The biology processes were considered to be significant with the thresholds of false discovery rate (FDR) < 0.05 as showed in Table 4. Hairs detach dermal papilla and stop growing since catagen, and then they become dull and lifeless at telogen because of no blood supply. For the only muscle in hair follicles, the arrector pilli muscles is not just an attacher to hair follicle–as had been reported earlier- but is an essential part of the hair follicle cycle by interacting with the follicle mesenchyme[20]. As expected, between catagen SHF and telogen SHF, the DEGs were related to muscle contraction (GO:0006936) and muscle filament sliding (GO:0030049) instead of growth and metabolism. Our results indicated that arrector pilli muscles, which attached to hair follicles, might have functions on helping cashmere stay in the hair follicles at catagen. Interestingly, the DEGs of PHF between the two phases of SHF were involved in those biology processes including translation (GO:0006412, 0006413, 0006414 and 0006415), RNA metabolism (GO:0016070 and 0016071), protein metabolism (GO:0044267), and viral growth (GO:0019083, 0019058 and 0016032). There is not any enriched GO category of the DEGs between PHF and SHF at the telogen phase of SHF, which is satisfied with the threshold at the telogen phase of SHF. In contrast, we found 22 enriched GO categories for the DEGs between the PHF at the catagen of SHF and catagen SHF, such as extracellular matrix organization (GO:0030198), muscle contraction (GO:0030049 and 0006936), translation (GO:0006413, 0006414, and 0006415), and metabolism (GO:0044281, 0006749, 0005975, and 0006805). All in all, these results might hint that the catagen phase of PHF might overlap with the catagen phase of SHF.

**Table 4:** GO annotation analysis.

GO ID	GO Term	DifGene	P-Value	FDR
PHF_catagen/PHF_telogen				
GO:0006414	translational elongation	82	7.16×10^−21^	1.76×10^−17^
GO:0019083	viral transcription	73	5.03×10^−20^	6.17×10^−17^
GO:0006415	translational termination	73	1.25×10^−19^	1.02×10^−16^
GO:0019058	viral life cycle	73	6.23×10^−19^	3.82×10^−16^
GO:0006614	SRP-dependent cotranslational protein targeting to membrane	73	2.54×10^−18^	1.06×10^−15^
GO:0006412	translation	101	2.60×10^−18^	1.06×10^−15^
GO:0000184	nuclear-transcribed mRNA catabolic process, nonsense-mediated decay	75	3.02×10^−18^	1.06×10^−15^
GO:0006413	translational initiation	78	7.87×10^−18^	2.41×10^−15^
GO:0016071	mRNA metabolic process	80	9.53×10^−15^	2.60×10^−12^
GO:0016070	RNA metabolic process	81	4.19×10^−14^	1.03×10^−11^
GO:0016032	viral process	87	4.09×10^−13^	9.12×10^−11^
GO:0044267	cellular protein metabolic process	104	3.53×10^−12^	7.23×10^−10^
GO:0010467	gene expression	120	3.85×10^−11^	7.26×10^−9^
GO:0042273	ribosomal large subunit biogenesis	11	3.64×10^−5^	6.39×10^−3^
SHF_catagen/SHF_telogen				
GO:0006936	muscle contraction	21	6.84×10^−11^	1.30×10^−7^
GO:0030049	muscle filament sliding	14	3.23×10^−10^	3.07×10^−7^
GO:0060048	cardiac muscle contraction	9	1.40×10^−5^	7.64×10^−3^
GO:0045214	sarcomere organization	8	1.61×10^−5^	7.64×10^−3^
SHF_catagen/PHF_catagen				
GO:0030198	extracellular matrix organization	64	1.32×10^−9^	3.69×10^−6^
GO:0030049	muscle filament sliding	23	1.88×10^−9^	3.69×10^−6^
GO:0006936	muscle contraction	32	1.73×10^−7^	2.27×10^−4^
GO:0006414	translational elongation	72	5.57×10^−7^	5.47×10^−4^
GO:0019083	viral transcription	62	1.55×10^−6^	1.22×10^−3^
GO:0019058	viral life cycle	64	2.00×10^−6^	1.31×10^−3^
GO:0006415	translational termination	62	2.64×10^−6^	1.48×10^−3^
GO:0030199	collagen fibril organization	17	4.45×10^−6^	2.19×10^−3^
GO:0044281	small molecule metabolic process	207	6.15×10^−6^	2.69×10^−3^
GO:0000184	nuclear-transcribed mRNA catabolic process, nonsense-mediated decay	65	1.08×10^−5^	4.26×10^−3^
GO:0006614	SRP-dependent cotranslational protein targeting to membrane	62	1.48×10^−5^	5.29×10^−3^
GO:0006749	glutathione metabolic process	14	2.94×10^−5^	9.62×10^−3^
GO:0060048	cardiac muscle contraction	16	3.19×10^−5^	9.64×10^−3^
GO:0006937	regulation of muscle contraction	11	5.96×10^−5^	1.67×10^−2^
GO:0006413	translational initiation	66	6.60×10^−5^	1.72×10^−2^
GO:0005975	carbohydrate metabolic process	73	6.99×10^−5^	1.72×10^−2^
GO:0001666	response to hypoxia	33	1.46×10^−4^	3.37×10^−2^
GO:1901687	glutathione derivative biosynthetic process	12	1.63×10^−4^	3.56×10^−2^
GO:0042060	wound healing	20	1.97×10^−4^	4.08×10^−2^
GO:0016071	mRNA metabolic process	74	2.11×10^−4^	4.14×10^−2^
GO:0007155	cell adhesion	92	2.35×10^−4^	4.27×10^−2^
GO:0006805	xenobiotic metabolic process	32	2.39×10^−4^	4.27×10^−2^
SHF_telogen/PHF_telogen				

Besides the GO analysis, we also enriched the KEGG pathways for DEGs with FDR < 0.05 in order to collect the molecular interaction, reaction and relation among DEGs. As same as the enriched biological processes in the GO analysis, the pathways for the DEGs between catagen SHF and telogen SHF are also associated with the function of muscle (Table 5). Furthermore, we also observed that the most pathways were observed for the DEGs between the PHF at the catagen of SHF and catagen SHF, including extracellular matrix interaction (PATH:04512 and 04510), metabolism (PATH:04974, 0006749, 00480, 00980 and 05240) and ribosome (PATH:03010). Against that several pathways for the comparisons as above described, the only enriched pathway for the DEGs of PHF between the two phases of SHF was ribosome (PATH:03010); besides, none can be enriched for the DEGs between PHF and SHF at the telogen phase of SHF.

**Table 5:** KEGG pathways analysis.

Pathway ID	Pathway Term	DifGene	P-Value	FDR
PHF_catagen/PHF_telogen				
PATH:03010	Ribosome	79	3.29×10^−18^	8.00×10^−16^
SHF_catagen/SHF_telogen				
PATH:04530	Tight junction	16	5.62×10^−5^	1.20×10^−2^
PATH:05410	Hypertrophic cardiomyopathy (HCM)	11	4.35×10^−4^	4.51×10^−2^
PATH:05414	Dilated cardiomyopathy	11	6.63×10^−4^	4.51×10^−2^
PATH:05416	Viral myocarditis	9	8.47×10^−4^	4.51×10^−2^
SHF_catagen/PHF_catagen				
PATH:04512	ECM-receptor interaction	29	2.27×10^−6^	6.09×10^−4^
PATH:04510	Focal adhesion	47	1.28×10^−5^	1.71×10^−3^
PATH:04974	Protein digestion and absorption	27	2.71×10^−5^	2.42×10^−3^
PATH:00480	Glutathione metabolism	18	1.19×10^−4^	6.92×10^−3^
PATH:03010	Ribosome	66	1.29×10^−4^	6.92×10^−3^
PATH:00980	Metabolism of xenobiotics by cytochrome P450	21	5.37×10^−4^	2.40×10^−2^
PATH:05134	Legionellosis	19	1.19×10^−3^	4.55×10^−2^
PATH:05204	Chemical carcinogenesis	18	1.36×10^−3^	4.55×10^−2^
SHF_telogen/PHF_telogen				

**Fig 3:**
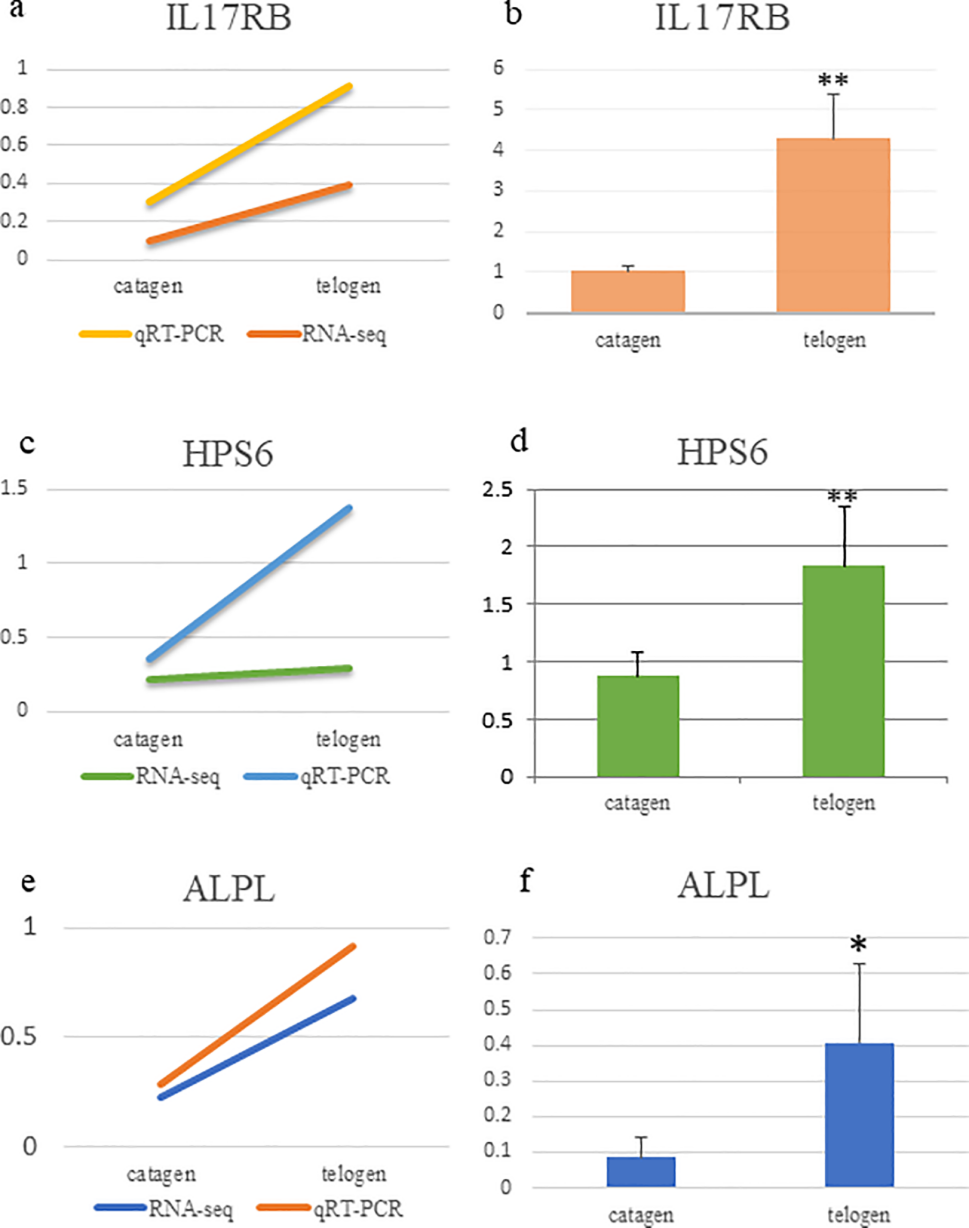
*IL17RB*, *HPS6* and *ALPL* was detected by the qRT-PCR, and the trend was similar to RNA-seq. ** p-value < 0.01, * p-value < 0.05.

According to GO terms, we made an examination using qRT-PCR for the DEGs, such as *IL17RB* (Interleukin 17 receptor B), *HPS6*(biogenesis of lysosomal organelles complex 2 subunit 3) and *ALPL*(alkaline phosphatase), which are functionally related to hair follicle growth Fig 3). The result showed that these three genes were upregulated in catagen SHF compared with telogen SHF, which is consistent with the result of RNA-seq. We also found that the DEGs between catagen SHF and telogen included eight members of zinc finger protein family (Table 6), of which seven members were upregulated except for *ZNF347*.

**Table 6:** DEGs from zinc finger protein family in SHF during catagen to telogen.

AccID	expression counts in catagen	expression counts in telogen	Blast Human AccID	gene name
GOAT_ENSP00000395277	5647	52510	NM_015037.3	*ZSWIM8*
GOAT_ENSP00000331462	5	70	NM_032651.1	*ZNF704*
GOAT_ENSBTAP00000001064	1547	44890	NM_022103.3	*ZNF667*
goat_GLEAN_10007887	10	18	NM_182594.2	*ZNF454*
goat_GLEAN_10012700	0	6	NM_017757.2	*ZNF407*
GOAT_ENSBTAP00000052171-D2	164	1	NM_032584.2	*ZNF347*
GOAT_ENSBTAP00000018112	20	26	NM_003417.4	*ZNF264*
goat_GLEAN_10011985	0	3	NM_024721.4	*ZFHX4*

## Discussion

In this study, we chose hair follicle as an alternative to skin in order to reveal the molecular mechanism of the growth cycle of Inner Mongolia Cashmere goat hair follicle. Compared with skin, hair follicle is beneficial to remove the signals of hair stem cells in skin and to eliminate some unexpected influence. To date, we are acquainted with the growth law of SHF in Inner Mongolia Cashmere goat but are completely at a loss for the time-based growth pattern of PHF yet. This study might be helpful to find the relationship between these two hair follicles by collecting PHF at the catagen/telogen phase of SHF. We uncovered that the distinction of gene expression are mainly associated with muscle movement between catagen SHF and telogen one. However, the growth cycle of PHF is not as same as SHF’s; at SHF’s catagen phase, PHF looks like stay in an active state because many metabolism-related genes were enriched(S1 Table). But, interestingly, PHF might be of the same inactive state as SHF at SHF’ telogen phase because no one GO category was identified for DEGs between PHF and SHF.

**Fig 4:**
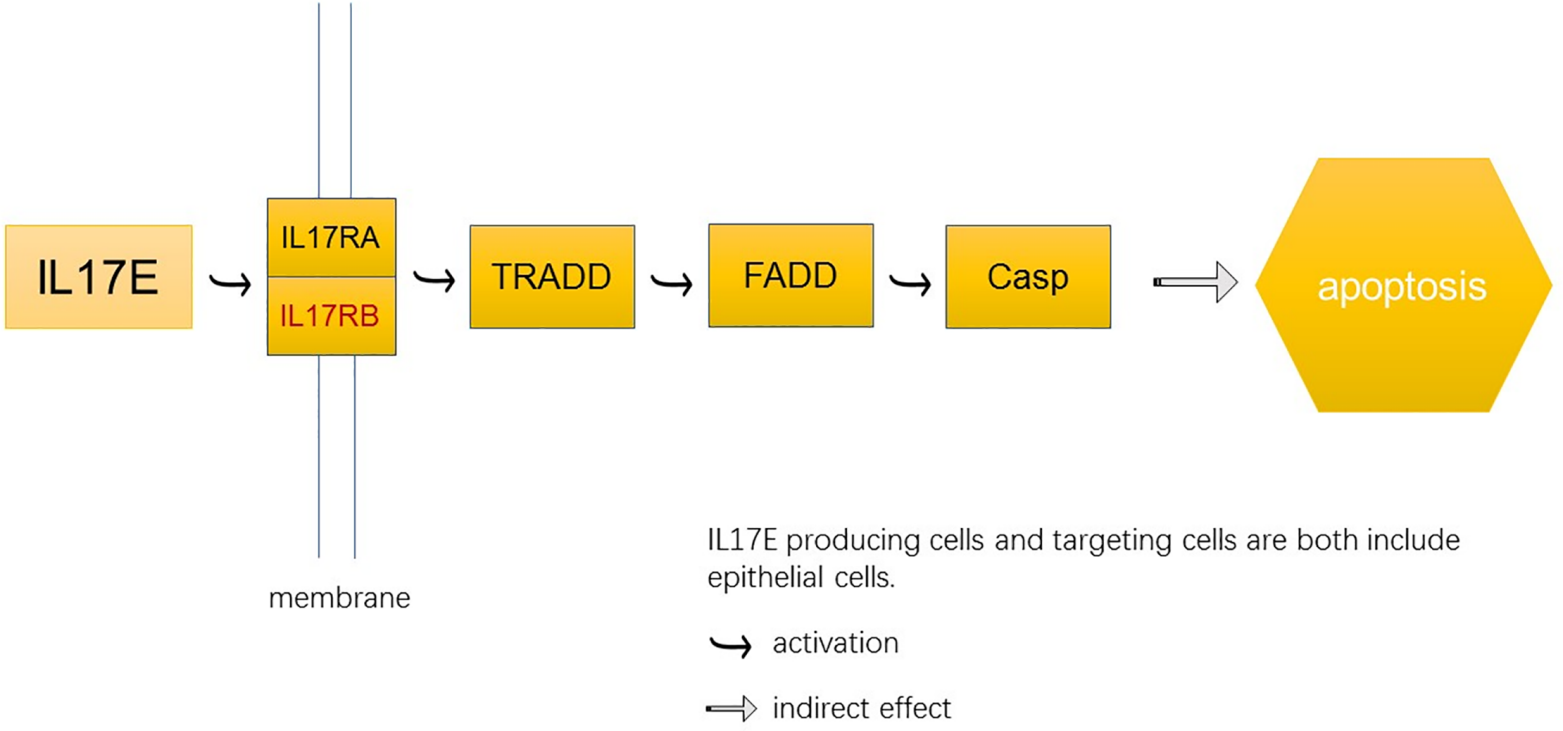
IL17RB-IL17E pathway may due to epithelial cells apoptosis.

In addition, we analyzed the expression pattern of several critical genes in SHF at the catagen- and telogen. We found that a critical gene *IL17RB* was upregulated in catagen SHF compared with telogen SHF. IL17 superfamily is a relatively new family of cytokines, which consists of 6 ligands and can bind 5 receptor subtypes[21]. According to recent researches and our transcriptome data, we predicted that *IL17RB* might play a role in the development of SHF because it acts as a mediate signaling in many other physiological functions[22,23]. A branch of IL-17 pathway, IL17E pathway, targets epithelial cell. The compound of *IL17RB* and *IL17RA* worked as membrane receptor signals of IL17E which would activate TRADD, FADD and Casp in order. Finally, this pathway may indirectly affect the apoptosis of epithelial cells (Fig 4). It might indicate that *IL17RB* plays a positive role in cell apoptosis, and then launches the transition from catagen to telogen. Also, two *IL17RB* can also form a compound which acted as a membrane receptor of *IL17B* in IL17RB-IL17B pathway. Vahideh Alinejad et al. found that the IL17RB-IL17B pathway affected breast cancer together with the upstream and downstream cytokines[24]. They summarized IL17 family includes six protein members—*IL17A*, *IL17B*, *IL17C*, *IL17D*, *IL17E* and *IL17F*, of which *IL17B* and its receptor(IL17RB) are least known genes[25,26].

Then, we identified that the eight members of zinc finger protein family were the DEGs between catagen SHF and telogen one (Table 6), of which seven members were upregulated except for *ZNF347*. Zinc finger proteins are important structural elements in many nucleic acid binding proteins [27]. The researchers further found that zinc finger proteins might affect the growth cycle of hair follicle [28,29]. A zinc finger transcription factor *Trps1* mainly expressed at the nuclei of mesenchymal cells during hair follicle morphogenesis; its expression level increased in dermis but decreased in epidermis during early skin morphogenesis [28]. In 2013, *Zfp157*, a member of KRAB zinc finger protein family, expressed in both epithelial cells of ducts and sebaceous glands of hair follicles in mice [29]. Notably, *ZNF667* can inhibit the expression and promoter activity of the rat proapoptotic gene *Bax* [30]. *ZNF667* was a DEG from catagen to telogen of both SHF and PHF in our study, this may indicate that *ZNF667* might be an important impact factor for the transition of SHF and PHF from catagen to telogen.

In our previous study, we found that *STC2* was downregulated from anagen to catagen[31]. Teng Xu et al. identified approximate 7,000 transcripts that were differentially expressed between anagen and telogen of Inner Mongolia Cashmere goat. In addition, the genes were mainly enriched in ECM receptor interaction, focal adhesion and gap junction from the KEGG pathway database[32]. Rongqing Geng et al. totally identified 1,332 DEGs in Shaanbei Cashmere goat among anagen, catagen and telogen and these genes were mainly playing important roles in Wnt, Shh, TGF-β, and Notch signaling pathways[8]. Xiao-yang Ji et al. found that ubiquitin-mediated proteolysis pathway is a prominent signaling pathway that can distinguish SHF from PHF in cashmere goats [33]. Jianping Li et al. found ten microRNAs have influence in hair follicle growth in Liaoning Cashmere goat and fine-wool sheep telogen skins [34].

The researchers also kept on excavating inhibitors that may affect the growth of hair follicle from model animals to cashmere goats. Plikus et al. indicated BMPs might be a potential signal that played as an inhibitor in the propagation of hair stem cells in mice[12]. Wen L. Bai et al. found that *BMP4* is generally upregulated from anagen to telogen in Liaoning Cashmere goat skin tissue, a higher *BMP4* methylation level in skin coincides with a lower expression of *BMP4* mRNA[35]. They also found seven downregulated miRNAs could joined some signal pathways through their target genes directly or indirectly from anagen to telogen[36]. Mathieu Castela et al. suggested *IGF1R* would together with *BMP4* to control hair follicle goes into anagen early and into catagen late using K15-IGF1RKO mice [37]. Xianghui Ma et al. found *MSI2*, a RNA-binding protein of Musashi, would keep hair follicle resting in process of telogen-to-anagen transition in mice[38]. In recent study, Guangxian Zhou et al. revealed ncRNA might play a regulatory role in skin of cashmere goat through whole transcriptome[39]. Our study might improve the understanding on the molecular mechanism of the growth cycle of SHF and PHF in Cashmere goats.

## Conclusion

In this study, we aim to find out about some interesting factors and signaling pathways, using transcriptome sequencing, that have functions on the growth of two kinds of hair in Inner Mongolia Cashmere goats at the catagen- and telogen phase of SHF. We totally found 1977 DEGs between PHF at the telogen and catagen phases of SHF, 1199 DEGs between telogen and catagen SHF, 2629 DEGs between catagen SHF and PHF at the catagen phase of SHF, and 755 DEGs between telogen SHF and PHF at the telogen phase of SHF. Between catagen- and telogen SHF, the DEGs were related to muscle contraction (GO:0006936) and muscle filament sliding (GO:0030049). Moreover, according to GO category of the DEGs between PHF and SHF, part of PHF might be in anagen phase at SHF’s catagen, but PHF might enter into telogen phase at SHF’s telogen. Among DEGs, *IL17RB* and eight members of ZNF might play important roles in the growth at the transition of hair follicles from catagen to telogen. These results would benefit the studies of the growth of two kinds of hair follicles in Inner Mongolia Cashmere goats.

## Supporting information

S1 Table Regulate style about metabolism-related genes in PHF_catagen/PHF_telogen. (DOCX)
