# Supplementary material for: Transcriptomic analysis reveals critical genes for the hair follicle of Inner Mongolia cashmere goat from catagen to telogen
Source: PLoS One. 2018 Oct 24;13(10):e0204404. doi: 10.1371/journal.pone.0204404 (PMC6200190; doi:10.1371/journal.pone.0204404)
Supplement: S1 Table — (DOCX) [file pone.0204404.s001.docx]

**S1 Table. Regulate style about metabolism-related genes in PHF_catagen/PHF_telogen.**

| **AccID** | **GO Term** | **style** |
| --- | --- | --- |
| GOAT_ENSBTAP00000003273 | cellular metabolic process | down |
| GOAT_ENSBTAP00000016013 | cellular metabolic process | down |
| GOAT_ENSBTAP00000016957 | cellular metabolic process | down |
| GOAT_ENSBTAP00000018778 | cellular metabolic process | up |
| GOAT_ENSBTAP00000025322 | cellular metabolic process | down |
| GOAT_ENSBTAP00000050729 | cellular metabolic process | up |
| GOAT_ENSBTAP00000051455 | cellular metabolic process | up |
| GOAT_ENSP00000007516 | cellular metabolic process | down |
| GOAT_ENSP00000369134 | cellular metabolic process | up |
| goat_GLEAN_10005558 | cellular metabolic process | down |
| goat_GLEAN_10008622 | cellular metabolic process | up |
| goat_GLEAN_10008729 | cellular metabolic process | down |
| goat_GLEAN_10012637 | cellular metabolic process | up |
| GOAT_ENSBTAP00000002603 | mRNA metabolic process RNA metabolic process | up |
| GOAT_ENSBTAP00000014565 | mRNA metabolic process RNA metabolic process | up |
| GOAT_ENSBTAP00000015883 | mRNA metabolic process RNA metabolic process | down |
| GOAT_ENSBTAP00000018910 | mRNA metabolic process RNA metabolic process | up |
| GOAT_ENSBTAP00000027632 | mRNA metabolic process RNA metabolic process | up |
| GOAT_ENSBTAP00000031700 | mRNA metabolic process RNA metabolic process | down |
| GOAT_ENSBTAP00000033015 | mRNA metabolic process RNA metabolic process | up |
| GOAT_ENSBTAP00000037041 | mRNA metabolic process RNA metabolic process | up |
| GOAT_ENSBTAP00000039829 | mRNA metabolic process RNA metabolic process | up |
| GOAT_ENSBTAP00000048426 | mRNA metabolic process RNA metabolic process | up |
| GOAT_ENSBTAP00000050222 | mRNA metabolic process RNA metabolic process | up |
| GOAT_ENSBTAP00000053003-D5 | mRNA metabolic process RNA metabolic process | up |
| GOAT_ENSP00000262563 | mRNA metabolic process RNA metabolic process | up |
| GOAT_ENSP00000296674 | mRNA metabolic process RNA metabolic process | up |
| GOAT_ENSP00000339064 | mRNA metabolic process RNA metabolic process | down |
| GOAT_ENSP00000346012 | mRNA metabolic process RNA metabolic process | down |
| GOAT_ENSP00000346022 | mRNA metabolic process RNA metabolic process | up |
| GOAT_ENSP00000351314 | mRNA metabolic process RNA metabolic process | down |
| GOAT_ENSP00000410758 | mRNA metabolic process RNA metabolic process | down |
| goat_GLEAN_10003610 | mRNA metabolic process RNA metabolic process | up |
| goat_GLEAN_10003782 | mRNA metabolic process RNA metabolic process | up |
| goat_GLEAN_10003802 | mRNA metabolic process RNA metabolic process | up |
| goat_GLEAN_10003937 | mRNA metabolic process RNA metabolic process | up |
| goat_GLEAN_10004095 | mRNA metabolic process RNA metabolic process | up |
| goat_GLEAN_10004152 | mRNA metabolic process RNA metabolic process | up |
| goat_GLEAN_10004450 | mRNA metabolic process RNA metabolic process | up |
| goat_GLEAN_10004636 | mRNA metabolic process RNA metabolic process | up |
| goat_GLEAN_10004814 | mRNA metabolic process RNA metabolic process | up |
| goat_GLEAN_10005280 | mRNA metabolic process RNA metabolic process | up |
| goat_GLEAN_10005473 | mRNA metabolic process RNA metabolic process | up |
| goat_GLEAN_10006186 | mRNA metabolic process RNA metabolic process | up |
| goat_GLEAN_10006595 | mRNA metabolic process RNA metabolic process | up |
| goat_GLEAN_10006648 | mRNA metabolic process RNA metabolic process | up |
| goat_GLEAN_10007185 | mRNA metabolic process RNA metabolic process | down |
| goat_GLEAN_10007212 | mRNA metabolic process RNA metabolic process | up |
| goat_GLEAN_10007819 | mRNA metabolic process RNA metabolic process | up |
| goat_GLEAN_10007820 | mRNA metabolic process RNA metabolic process | up |
| goat_GLEAN_10007932 | mRNA metabolic process RNA metabolic process | up |
| goat_GLEAN_10008997 | mRNA metabolic process RNA metabolic process | up |
| goat_GLEAN_10009015 | mRNA metabolic process RNA metabolic process | up |
| goat_GLEAN_10009076 | mRNA metabolic process RNA metabolic process | up |
| goat_GLEAN_10009481 | mRNA metabolic process RNA metabolic process | up |
| goat_GLEAN_10009625 | mRNA metabolic process RNA metabolic process | up |
| goat_GLEAN_10009760 | mRNA metabolic process RNA metabolic process | up |
| goat_GLEAN_10009846 | mRNA metabolic process RNA metabolic process | up |
| goat_GLEAN_10010125 | mRNA metabolic process RNA metabolic process | up |
| goat_GLEAN_10010314 | mRNA metabolic process RNA metabolic process | up |
| goat_GLEAN_10010802 | mRNA metabolic process RNA metabolic process | down |
| goat_GLEAN_10011726 | mRNA metabolic process RNA metabolic process | up |
| goat_GLEAN_10011820 | mRNA metabolic process RNA metabolic process | up |
| goat_GLEAN_10012260 | mRNA metabolic process RNA metabolic process | up |
| goat_GLEAN_10012476 | mRNA metabolic process RNA metabolic process | up |
| goat_GLEAN_10013044 | mRNA metabolic process RNA metabolic process | up |
| goat_GLEAN_10013075 | mRNA metabolic process RNA metabolic process | up |
| goat_GLEAN_10013171 | mRNA metabolic process RNA metabolic process | up |
| goat_GLEAN_10013222 | mRNA metabolic process RNA metabolic process | up |
| goat_GLEAN_10013274 | mRNA metabolic process RNA metabolic process | up |
| goat_GLEAN_10013548 | mRNA metabolic process RNA metabolic process | up |
| goat_GLEAN_10013723 | mRNA metabolic process RNA metabolic process | up |
| goat_GLEAN_10014480 | mRNA metabolic process RNA metabolic process | up |
| goat_GLEAN_10016553 | mRNA metabolic process RNA metabolic process | up |
| goat_GLEAN_10017197 | mRNA metabolic process RNA metabolic process | down |
| goat_GLEAN_10017255 | mRNA metabolic process RNA metabolic process | up |
| goat_GLEAN_10017282 | mRNA metabolic process RNA metabolic process | up |
| goat_GLEAN_10017557 | mRNA metabolic process RNA metabolic process | up |
| goat_GLEAN_10017694 | mRNA metabolic process RNA metabolic process | up |
| goat_GLEAN_10017719 | mRNA metabolic process RNA metabolic process | up |
| goat_GLEAN_10017954 | mRNA metabolic process RNA metabolic process | down |
| goat_GLEAN_10018534 | mRNA metabolic process RNA metabolic process | down |
| goat_GLEAN_10018855 | mRNA metabolic process RNA metabolic process | up |
| goat_GLEAN_10018879 | mRNA metabolic process RNA metabolic process | up |
| goat_GLEAN_10019009 | mRNA metabolic process RNA metabolic process | up |
| goat_GLEAN_10019219 | mRNA metabolic process RNA metabolic process | up |
| goat_GLEAN_10019528 | mRNA metabolic process RNA metabolic process | up |
| goat_GLEAN_10019597 | mRNA metabolic process RNA metabolic process | up |
| goat_GLEAN_10020036 | mRNA metabolic process RNA metabolic process | up |
| goat_GLEAN_10020080 | mRNA metabolic process RNA metabolic process | up |
| goat_GLEAN_10020719 | mRNA metabolic process RNA metabolic process | up |
| goat_GLEAN_10020750 | mRNA metabolic process RNA metabolic process | up |
| goat_GLEAN_10021061 | mRNA metabolic process RNA metabolic process | up |
| GOAT_ENSBTAP00000007083 | RNA metabolic process | up |
| goat_GLEAN_10004450 | RNA metabolic process | up |
